# Supplementary material for: Cryo-EM structure of the essential ribosome assembly AAA-ATPase Rix7
Source: Nat Commun. 2019 Jan 31;10:513. doi: 10.1038/s41467-019-08373-0 (PMC6355894; doi:10.1038/s41467-019-08373-0)
Supplement: Supplementary file 1 — Supplementary Information [file 41467_2019_8373_MOESM1_ESM.pdf]

Supplementary information

Cryo-EM Structure of the Essential Ribosome Assembly AAA-ATPase Rix7

Lo et al.

Supplementary Figure 1

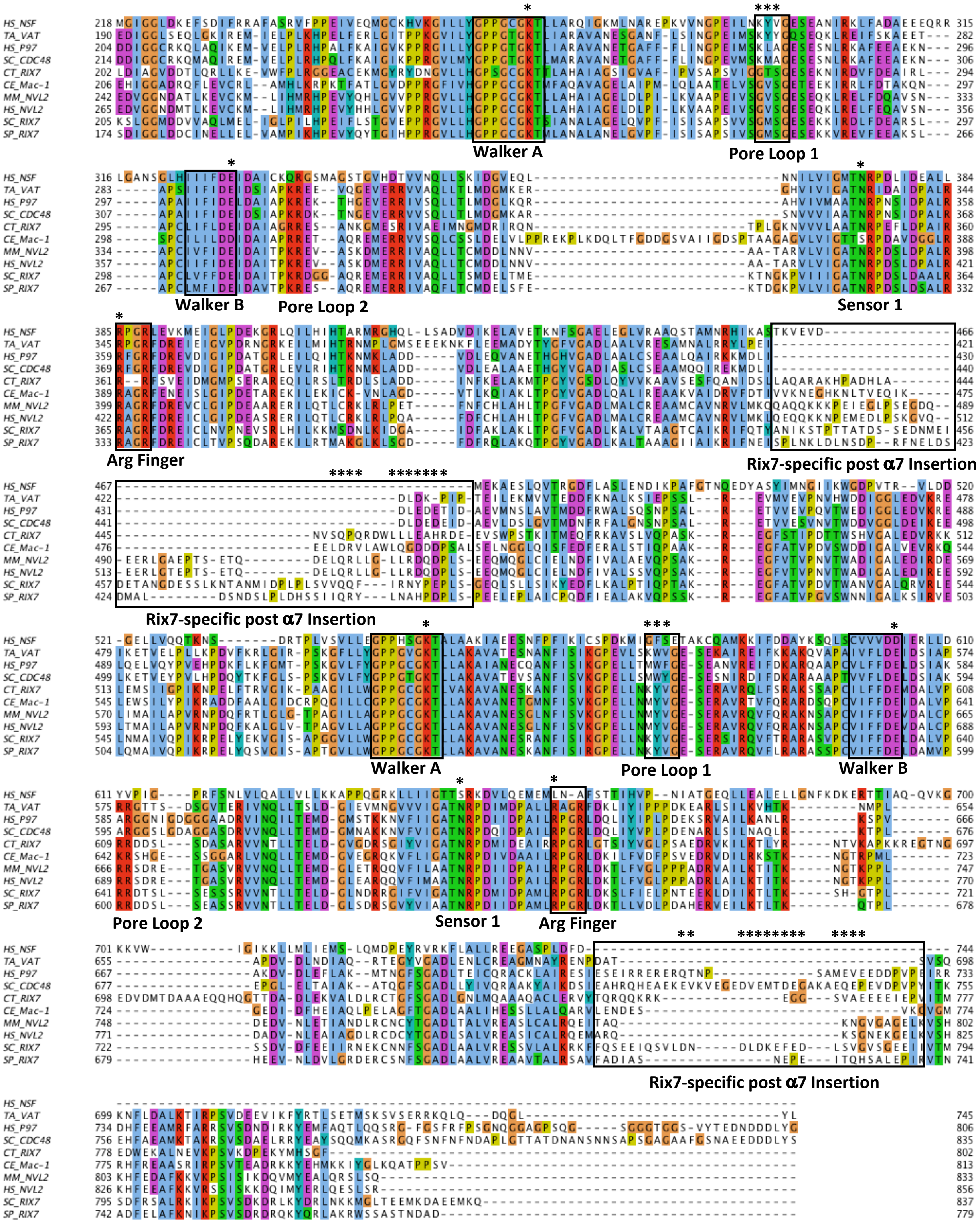

Supplementary Figure 1. Multiple sequence alignment of the D1 and D2 domains from several type II AAA family members, including NSF, VAT/p97/Cdc48, and Rix7/NVL2/Mac-1. Abbreviations are as follows: *Homo sapiens* (HS), *Thermoplasma acidophilum* (TA), *S. cerevisiae* (SC), *Chaetomium thermophilum* (CT), *Caenorhabditis elegans* (CE), *Mus Musculus* (MM) and *Schizosaccharomyces pombe* (SP). Alignments were done in Clustal omega[1] and illustrated with JalView[2]. Motifs important for ATP binding and hydrolysis are labeled below the sequence. The Walker A motifs, Walker B motifs, post $\alpha 7$ insertions, arginine finger, and pore loop 1 regions are further indicated with a black box. Residues targeted for mutagenesis and deletion are marked with asterisks.

Supplementary Figure 2

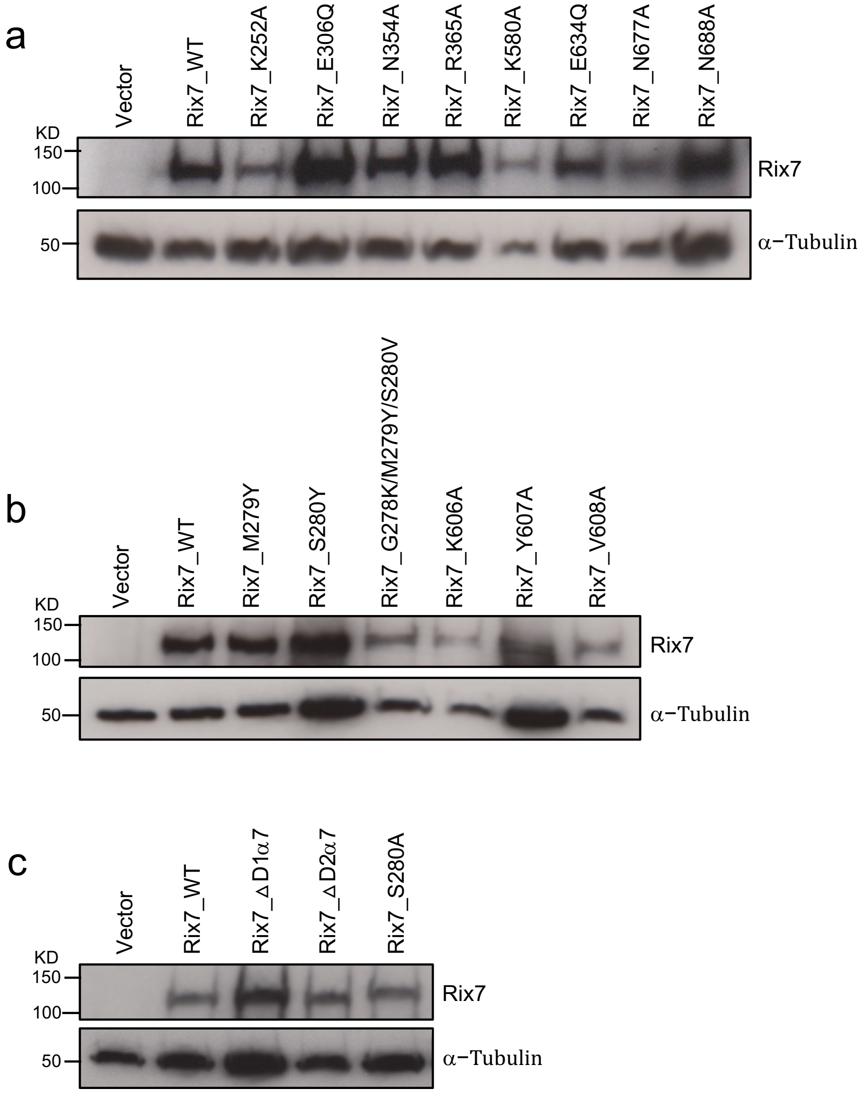

Supplementary Figure 2. Rix7 mutants are expressed in *S. cerevisiae.* Western blots showing expression of the all the Rix7 mutants. Rix7 variants were detected with a Flag tag antibody and α-tubulin was used as the loading control. **a** Rix7 mutants in two ATPase domains. **b** Rix7 mutants in pore loop 1 regions. **c** Rix7 mutant S280A and deletion in Rix7-specific post α7 insertion in both ATPase domains.

Supplementary Figure 3

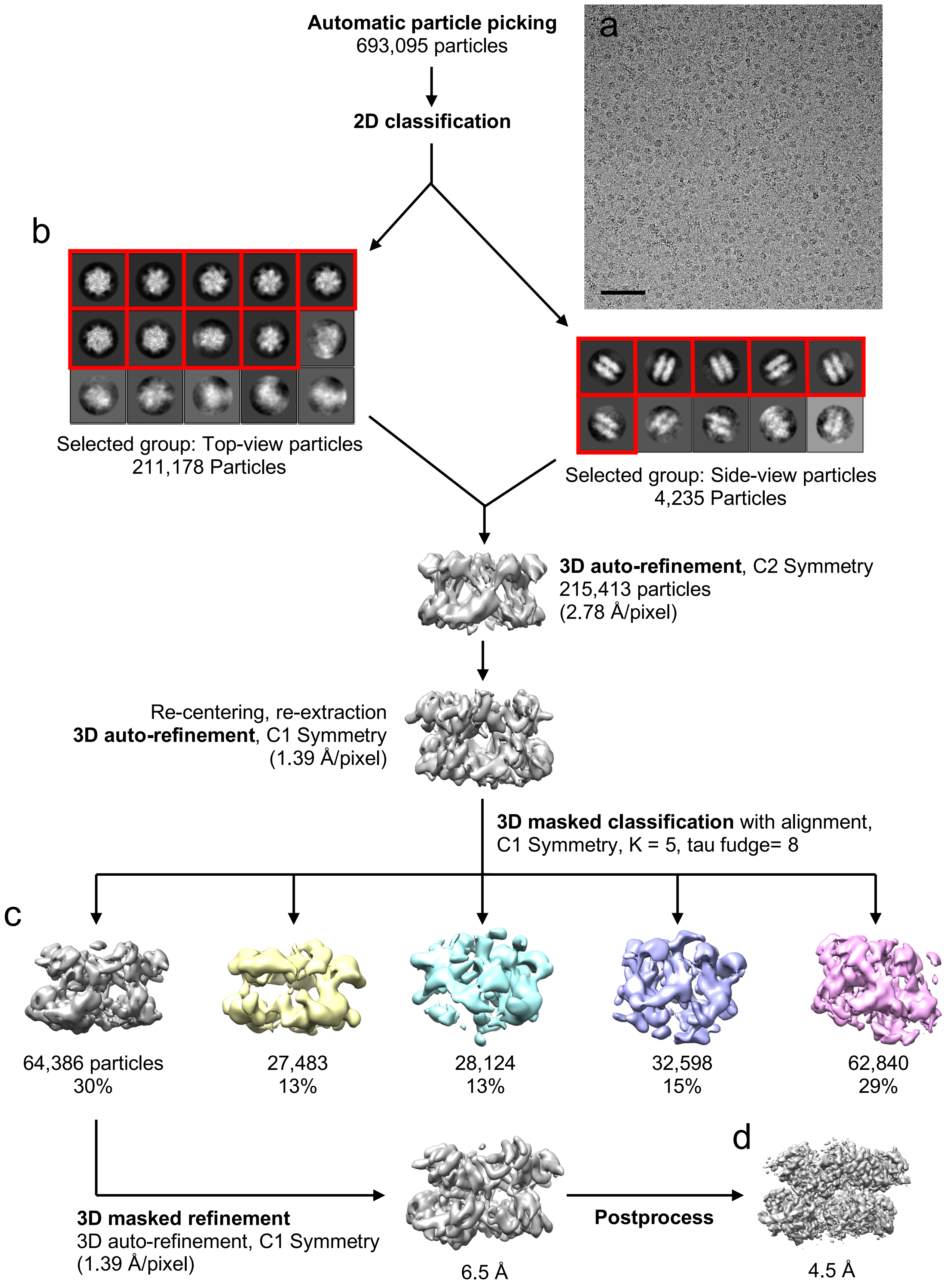

Supplementary Figure 3. Workflow of cryo-EM data processing. a Representative micrograph of Rix7 particles. A 60 nm scale bar is shown in the lower left-hand corner. **b** Representative 2D class averages from RELION[3]. Due to an orientation preference and overfitting, 2D classification for the top-view and the side-view were performed separately. Selected class averages (red box) were used to create an initial model generated in EMAN2[4]. Two rounds of refinement were performed, first with C2 symmetry and then without imposing symmetry. **c** 3D classes generated from RELION without imposing symmetry. The first class (shown in grey), which included the highest number of particles and estimated resolution, was used for further 3D refinement. **d** Final cryo-EM electron scattering density map determined using RELION post_processing[3].

Supplementary Figure 4

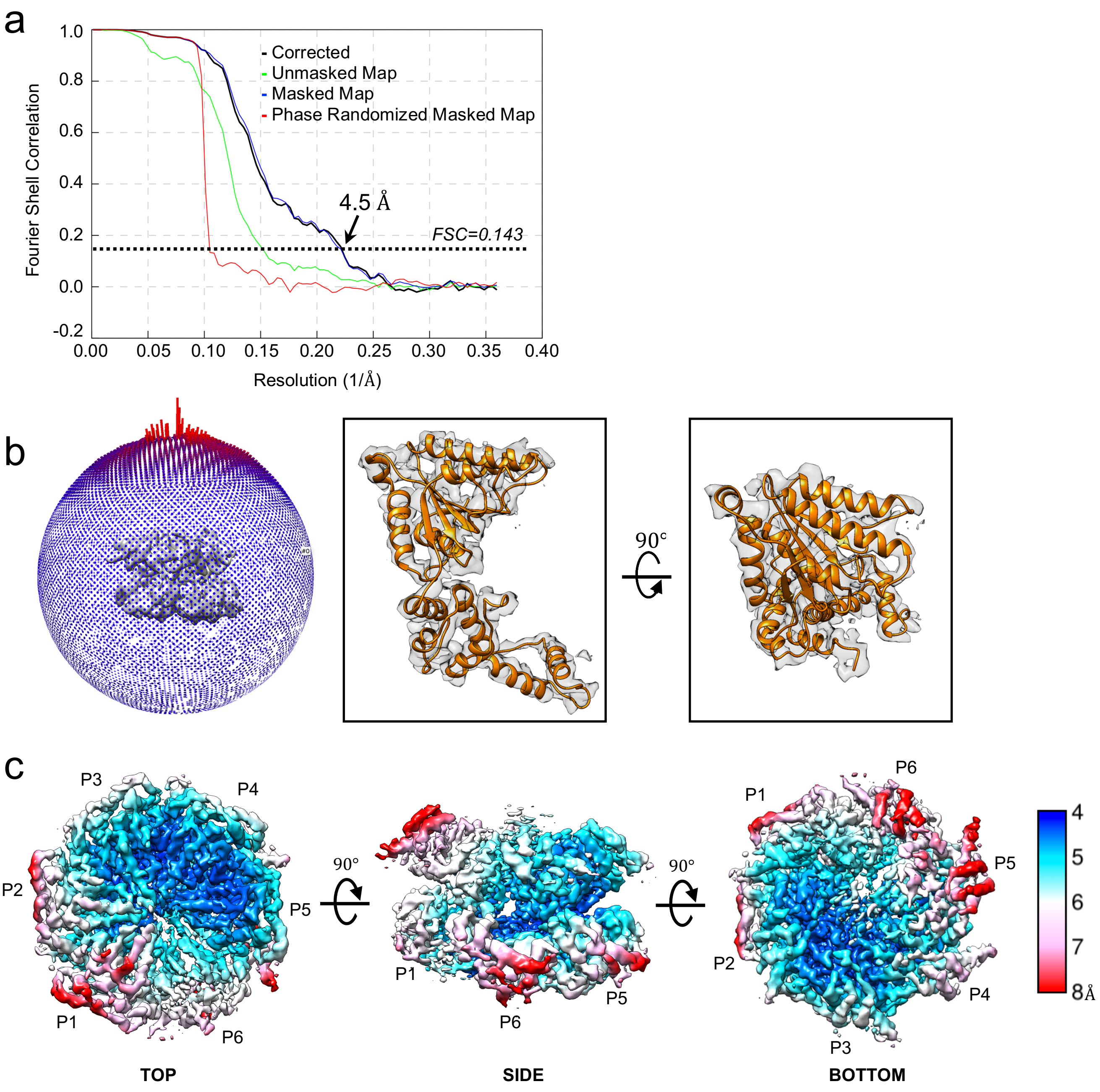

Supplementary Figure 4. Validation of the Rix7 reconstruction. a Standard FSC curve for the final model determined using RELION post_processing[3] indicates an estimated resolution of 4.5 Å. **b** Angular distribution of particles for the final refinement is shown with the corresponding view of the map (left). The number of particles with different orientation is indicated by color (blue=low, red=high) and size of the bar. The angular distribution shows that there are preferred orientations around Rix7 particles, however the resolution is not anisotropic, as demonstrated by the different views from the side (middle) and top (right) of Rix7 protomer 3. **c** Local resolution shown across the final 3D map for the reconstruction of Rix7 determined by Resmap[5].

Supplementary Figure 5

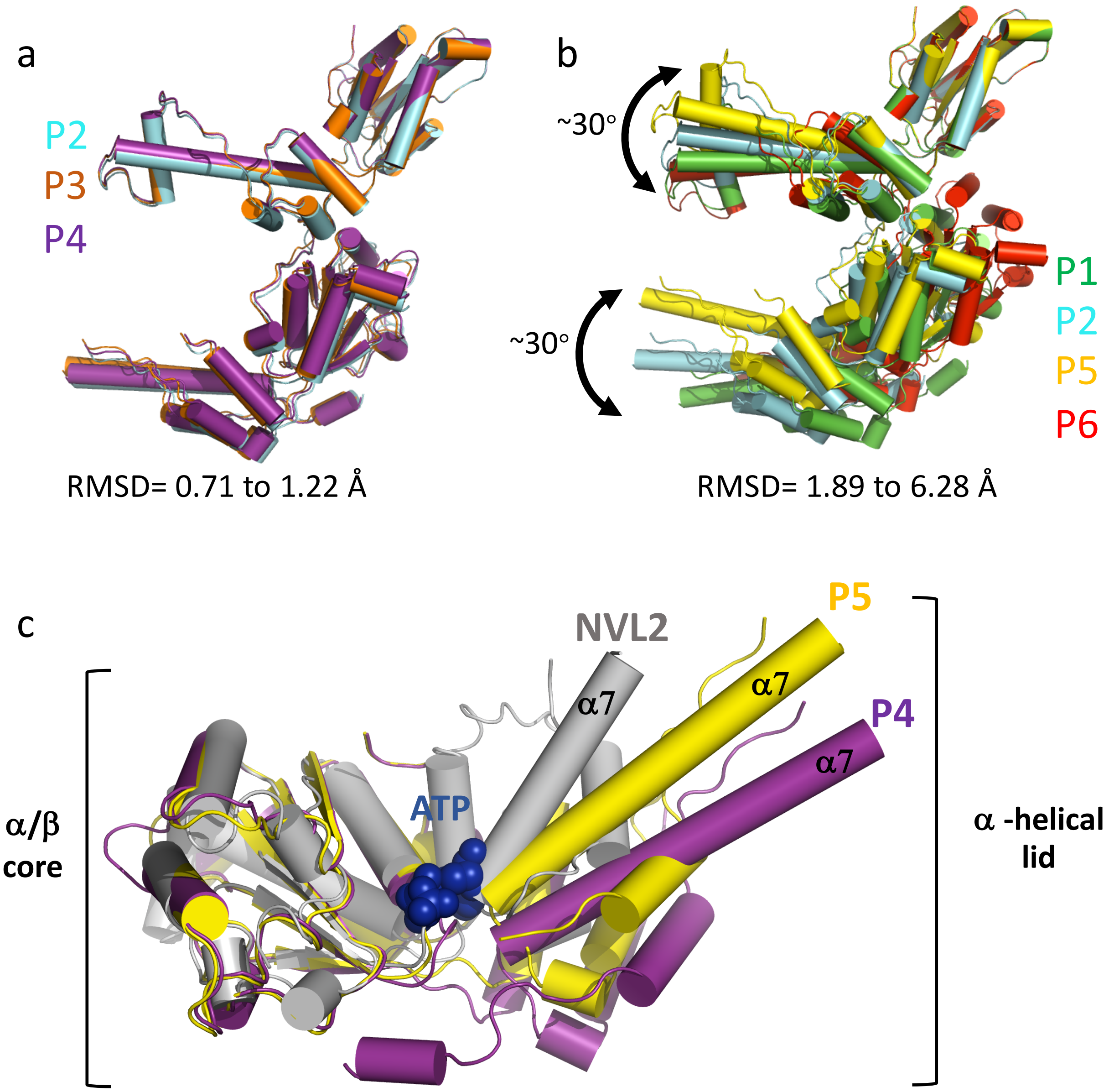

Supplementary Figure 5. The individual promoters of Rix7 are distinct from one another. Individual promoters colored as indicated. Each promoter was aligned on the α/β subdomain from the D1 domain. **a** Superposition of P2, P3, and P4 protomers. **b** Superposition of P1, P2, P5, and P6 protomers. **c** Overlay of the D2 AAA domain of the Rix7 P4 and P5 protomers with the NVL2 D2 domain (PDB ID 2X8A) which illustrates the conformational changes in α7 from P5 and NVL2 that block ATP binding.

Supplementary Figure 6

Figure S2a

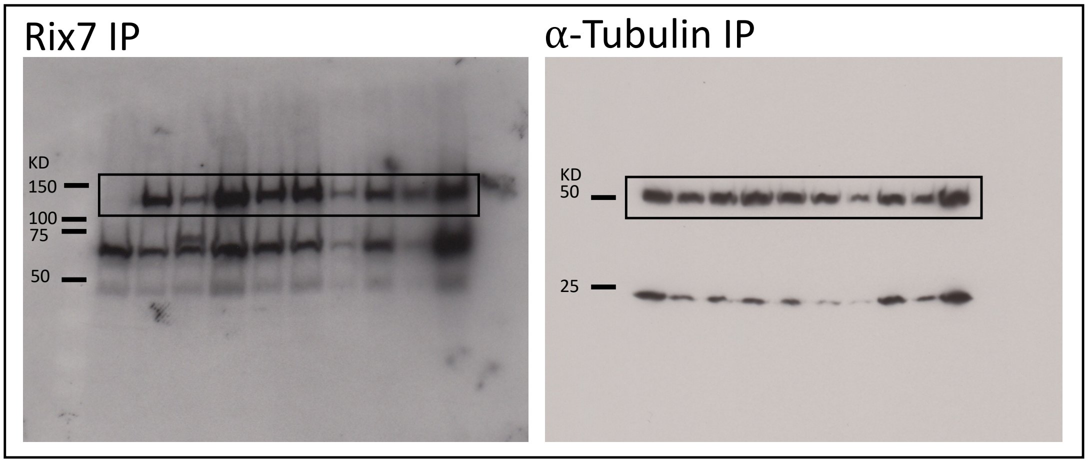

Figure S2b

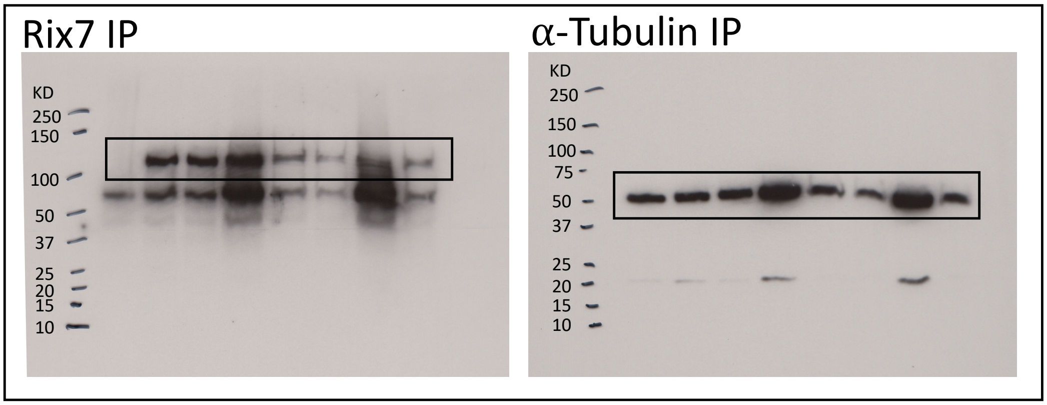

Figure S2c

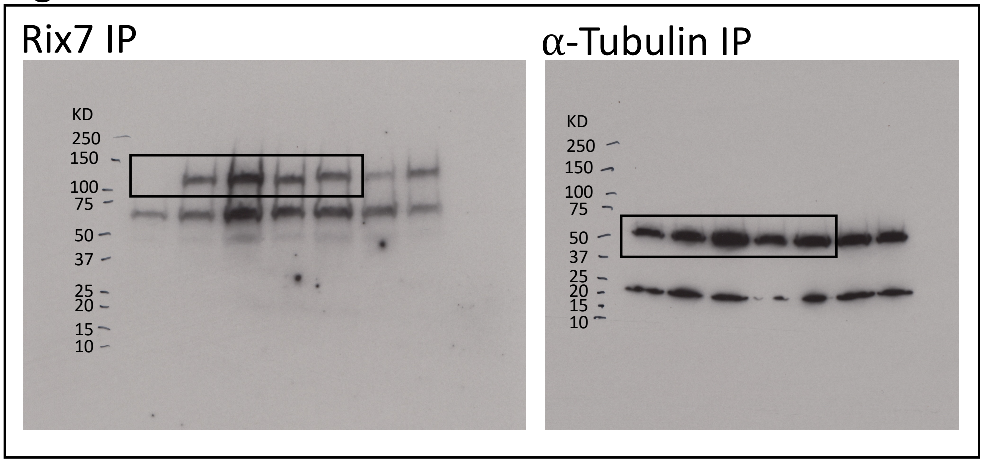

Supplementary Figure 6. Uncropped western blots from supplementary Figure 2. Boxes highlight area used for cropping.

Supplementary Table 1. Primers used in this study

Primer name	Primer sequence
Rix7-promotor-Fw	TGATTACGCCAAGCTTGCTTTTGCTGTTGTTATCGTAATAGAAGGC
Rix7-promotor-Rv	GTGAATTCGAGCTCGGTACCGTGACTTTCTTCCATCCCCCC
Rix7-Nter3xFlag-Fw	GATGATGATAAAGATTATAAAGATGATGATGATAAAGTTAAAGTAAAGTCGAAAAAGAAC
Rix7-Nter3xFlag-Rv	ATCTTTATAATCTTTATCATCATCATCTTTATAATCCATTATTCCAGCCTTCCC
Rix7-CterStrep-Fw	GTTTGAAAAATAAAAATTTATAAATAATATTTTGGTAAAGTC
Rix7_CterStrep-Rv	TGCGGATGGCTCCATTGTTTCATTTCTTCCGC
Rix7-K252A-Fw	GGGTTGCGGTGCGACGTCGATTGC
Rix7-K252A-Rv	GGTGGGCCATGCAACAGA
Rix7-E306Q-Fw	TTTCTTTGATCAAATAGATGCCATTAC
Rix7-E306Q-Rv	ACAAGACATGGTGCCAAG
Rix7-N354A-Fw	AGGTGCCACGGCAAGACCTGACTC
Rix7-N354A-Rv	ATGATAATAACAGGTTTTCCATTTG
Rix7-R365A-Fw	AGCATTGAGAGCAGCAGGTAGATTC
Rix7-R365A-Rv	GCATCCAAAGAGTCAGGTC
Rix7-K580A-Fw	AGGTTGTGGTGCAGCAACGTTACTAGCAAAAGC
Rix7-K580A-Rv	GGTGGTCCCCACAATAAAAC
Rix7-E634Q-Fw	ATTTTTTGATCAACTGGACGCTTTG
Rix7-E634Q-Rv	ATAACACACGGTACAGAC
Rix7-N677A-Fw	TGGCGCCACTGCAAGACCTGATATG
Rix7-N677A-Rv	ATCACAAAAATTCCCCTTCTATC
Rix7-R688A-Fw	TGCTATGTTAGCACCGGGCAGATTG
Rix7-R688A-Rv	GGATCAATCATATCAGGTCTATTAG
Rix7-M279Y-Fw	CGTAAGTGGTTATTCTGGGGAAAGCG
Rix7-M279Y-Rv	ACTGAAGGTGCAGAAATAG
Rix7-S280Y-Fw	AAGTGGTATGTATGGGGAAAGCG
Rix7-S280Y-Rv	ACGACTGAAGGTGCAGAAATAG
Rix7-S280A-Fw	AAGTGGTATGGCTGGGGAAAGCG
Rix7-S280A-Rv	ACGACTGAAGGTGCAGAAATAGATATAAATG
Rix7-G278K/M279Y/S280V-Fw	TGTTGGGGAAAGCGAGAAAAAG
Rix7-G278K/M279Y/S280V-Rv	TATTTACTTACGACTGAAGGTGC
Rix7-K606A-Fw	ATTATTAAATGCATACGTTGGTGAATCAG
Rix7-K606A-Rv	TCAGGACCTTTGATTGATATG
Rix7-Y607A-Fw	ATTAAATAAAGCCGTTGGTGAATCAGAAAG
Rix7-Y607A-Rv	AATTCAGGACCTTTGATTG
Rix7-V608A-Fw	AAATAAATACGCTGGTGAATCAG
Rix7-V608A-Rv	AATAATTCAGGACCTTTGATTG
Rix7-del-D1α7-Fw	GAGCCTTTATCAGGAGAG
Rix7-del-D1α7-Rv	TACGGATAAAGGCAACGG
Rix7-del-D2α7-Fw	GTATCTGGCGAAGAAATTATTG
Rix7-del-D2α7-Rv	AAGAACAGATTGTATTTCTTCG

Supplementary Table 2. Plasmids used in this study

Plasmid	Experiment		Vector	Source
Rix7 Ycplac111	Growth Assay	WT; residues 1-837	YCplac111	This study
Rix7 WT	Growth Assay/Sucrose gradient	WT; residues 1-837, 3X N-terminal FlagTag. C-terminal Strep Tag	YCplac111	This study
Rix7 D1WA	Growth Assay/ Sucrose gradient	D1 Walker A (K252A); residues 1-837, 3X N-terminal FlagTag. C-terminal Strep Tag	YCplac111	This study
Rix7 D1WB	Growth Assay/ Sucrose gradient	D1 Walker B (E306Q); residues 1-837, 3X N-terminal FlagTag. C-terminal Strep Tag	YCplac111	This study
Rix7 D1S1	Growth Assay	D1 Sensor 1 (N354A); residues 1-837, 3X N-terminal FlagTag. C-terminal Strep Tag	YCplac111	This study
Rix7 D1RF	Growth Assay	D1 Arginine Finder (R365E/A); residues 1-837, 3X N-terminal FlagTag. C-terminal Strep Tag	YCplac111	This study
Rix7 D2WA	Growth Assay/ Sucrose gradient	D2 Walker A(K580A); residues 1-837, 3X N-terminal FlagTag. C-terminal Strep Tag	YCplac111	This study
Rix7 D2WB	Growth Assay/ Sucrose gradient	D2 Walker B (E634Q); residues 1-837, 3X N-terminal FlagTag. C-terminal Strep Tag	YCplac111	This study
Rix7 D2S1	Growth Assay	D2 Sensor 1 (N677A); residues 1-837, 3X N-terminal FlagTag. C-terminal Strep Tag	YCplac111	This study
Rix7 D2RF	Growth Assay	D2 Arginine Finder (R688A); residues 1-837, 3X N-terminal FlagTag. C-terminal Strep Tag	YCplac111	This study
Rix7 D1-M279Y	Growth Assay	D1 Pore Loop M279Y; residues 1-837, 3X N-terminal FlagTag. C-terminal Strep Tag	YCplac111	This study
Rix7 D1-S280Y	Growth Assay	D1 Pore Loop S280Y; residues 1-837, 3X N-terminal FlagTag. C-terminal Strep Tag	YCplac111	This study
Rix7 D1-S280A	Growth Assay	D1 Pore Loop S280A; residues 1-837, 3X N-terminal FlagTag. C-terminal Strep Tag	YCplac111	This study
Rix7 D1-RYV	Growth Assay	D1 Pore Loop RYV (G278K/M279Y/S280V); residues 1-837, 3X N-terminal FlagTag. C-terminal Strep Tag	YCplac111	This study
Rix7 D2- K606A	Growth Assay	D2 Pore Loop K606A; residues 1-837, 3X N-terminal FlagTag. C-terminal Strep Tag	YCplac111	This study
Rix7 D2- Y607A	Growth Assay	D2 Pore Loop Y607A; residues 1-837, 3X N-terminal FlagTag. C-terminal Strep Tag	YCplac111	This study
Rix7 D2- V608A	Growth Assay	D2 Pore Loop V608A; residues 1-837, 3X N-terminal FlagTag. C-terminal Strep Tag	YCplac111	This study
Rix7 ΔD1α7	Growth Assay	ΔD1a7; residues 1-837$^{\Delta 482-492}$, 3X N-terminal FlagTag. C-terminal Strep Tag	YCplac111	This study
Rix7 ΔD2α7	Growth Assay	ΔD1a7; residues 1-837$^{\Delta 771-784}$, 3X N-terminal FlagTag. C-terminal Strep Tag	YCplac111	This study
CT-Rix7 D1/D2 WB	Protein Expression/cryo-EM analysis	*C. Thermophilum* Rix7 D1/D2 Walker B (E303Q/E602Q); 1-802, C-terminal 6 x His Tag	Pet24b	GenScript

Supplementary Table 3. Yeast strains used and constructed in this study

Strain	Genotype	Source
tetO$_7$-RIX7	*URA::CMV-tTA RIX7::kanR-tetO$_7$-TATA MATa his3-1 leu2-0 met15-0*	Tet-Promoter Hughes Collection (GE Dharmacon)
tetO$_7$-RIX7 NSA1-Flag	*URA::CMV-tTA RIX7::kanR-tetO7-TATA NSA1-5XFlag: MATa his3-1 leu2-0 met15-0*	This Study
tetO$_7$-RIX7 NSA1-Flag Rix7 WT	*URA::CMV-tTA RIX7::kanR-tetO7-TATA NSA1-5XFlag: MATa his3-1 leu2-0 met15-0; Rix7 WT*	This Study
tetO$_7$-RIX7 NSA1-Flag +Rix7 D1WA	*URA::CMV-tTA RIX7::kanR-tetO7-TATA NSA1-5XFlag: MATa his3-1 leu2-0 met15-0; Rix7 D1WA*	This Study
tetO$_7$-RIX7 NSA1-Flag +Rix7 D1WB	*URA::CMV-tTA RIX7::kanR-tetO7-TATA NSA1-5XFlag: MATa his3-1 leu2-0 met15-0; Rix7 D1WB*	This Study
tetO$_7$-RIX7 NSA1-Flag +Rix7 D1S1	*URA::CMV-tTA RIX7::kanR-tetO7-TATA NSA1-5XFlag: MATa his3-1 leu2-0 met15-0; Rix7 D1S1*	This Study
tetO$_7$-RIX7 NSA1-Flag +Rix7 D1RF	*URA::CMV-tTA RIX7::kanR-tetO7-TATA NSA1-5XFlag: MATa his3-1 leu2-0 met15-0; Rix7 D1RF*	This Study
tetO$_7$-RIX7 NSA1-Flag +Rix7 D2WA	*URA::CMV-tTA RIX7::kanR-tetO7-TATA NSA1-5XFlag: MATa his3-1 leu2-0 met15-0; Rix7 D2WA*	This Study
tetO$_7$-RIX7 NSA1-Flag +Rix7 D2WB	*URA::CMV-tTA RIX7::kanR-tetO7-TATA NSA1-5XFlag: MATa his3-1 leu2-0 met15-0; Rix7 D2WB*	This Study
tetO$_7$-RIX7 NSA1-Flag +Rix7 D2S1	*URA::CMV-tTA RIX7::kanR-tetO7-TATA NSA1-5XFlag: MATa his3-1 leu2-0 met15-0; Rix7 D2S1*	This Study
tetO$_7$-RIX7 NSA1-Flag +Rix7 D2RF	*URA::CMV-tTA RIX7::kanR-tetO7-TATA NSA1-5XFlag: MATa his3-1 leu2-0 met15-0; Rix7 D2RF*	This Study
tetO$_7$-RIX7 NSA1-Flag +Rix7 D1-M279Y	*URA::CMV-tTA RIX7::kanR-tetO7-TATA NSA1-5XFlag: MATa his3-1 leu2-0 met15-0; Rix7* D1-M279Y	This Study
tetO$_7$-RIX7 NSA1-Flag +Rix7 D1-S280Y	*URA::CMV-tTA RIX7::kanR-tetO7-TATA NSA1-5XFlag: MATa his3-1 leu2-0 met15-0; Rix7* D1-S280Y	This Study
tetO$_7$-RIX7 NSA1-Flag +Rix7 D1-S280A	*URA::CMV-tTA RIX7::kanR-tetO7-TATA NSA1-5XFlag: MATa his3-1 leu2-0 met15-0; Rix7* D1-S280A	This Study
tetO$_7$-RIX7 NSA1-Flag +Rix7 Rix7 D1-RYV	*URA::CMV-tTA RIX7::kanR-tetO7-TATA NSA1-5XFlag: MATa his3-1 leu2-0 met15-0; Rix7* D1-RYV	This Study
tetO$_7$-RIX7 NSA1-Flag +Rix7 D2-K606A	*URA::CMV-tTA RIX7::kanR-tetO7-TATA NSA1-5XFlag: MATa his3-1 leu2-0 met15-0; Rix7* D2- D2-K606A	This Study
tetO$_7$-RIX7 NSA1-Flag +Rix7 D2-Y607A	*URA::CMV-tTA RIX7::kanR-tetO7-TATA NSA1-5XFlag: MATa his3-1 leu2-0 met15-0; Rix7* D2-Y607A	This Study
tetO$_7$-RIX7 NSA1-Flag +Rix7 D2-V608A	*URA::CMV-tTA RIX7::kanR-tetO7-TATA NSA1-5XFlag: MATa his3-1 leu2-0 met15-0; Rix7* D2-V608A	This Study
tetO$_7$-RIX7 NSA1-Flag +Rix7 ΔD1α7	*URA::CMV-tTA RIX7::kanR-tetO7-TATA NSA1-5XFlag: MATa his3-1 leu2-0 met15-0; Rix7* ΔD1α7	This Study
tetO$_7$-RIX7 NSA1-Flag +Rix7 ΔD2α7	*URA::CMV-tTA RIX7::kanR-tetO7-TATA NSA1-5XFlag: MATa his3-1 leu2-0 met15-0; Rix7* ΔD2α7	This Study

Supplementary References

1. Sievers, F. et al. Fast, scalable generation of high-quality protein multiple sequence alignments using Clustal Omega. *Mol Syst Biol* **7**, 539 (2011).
2. Waterhouse, A.M., Procter, J.B., Martin, D.M., Clamp, M. & Barton, G.J. Jalview Version 2--a multiple sequence alignment editor and analysis workbench. *Bioinformatics* **25**, 1189-91 (2009).
3. Scheres, S.H. RELION: implementation of a Bayesian approach to cryo-EM structure determination. *J Struct Biol* **180**, 519-30 (2012).
4. Tang, G. et al. EMAN2: an extensible image processing suite for electron microscopy. *J Struct Biol* **157**, 38-46 (2007).
5. Kucukelbir, A., Sigworth, F.J. & Tagare, H.D. Quantifying the local resolution of cryo-EM density maps. *Nat Methods* **11**, 63-5 (2014).
