# Supplementary material for: Cryo-EM structure of the essential ribosome assembly AAA-ATPase Rix7
Source: Nat Commun. 2019 Jan 31;10:513. doi: 10.1038/s41467-019-08373-0 (PMC6355894; doi:10.1038/s41467-019-08373-0)
Supplement: Supplementary file 4 — Reporting Summary Checklist [file 41467_2019_8373_MOESM4_ESM.pdf]

naturereseach

Corresponding author(s): Robin E. Stanley

Reporting Summary

Nature Research wishes to improve the reproducibility of the work that we publish. This form provides structure for consistency and transparency in reporting. For further information on Nature Research policies, see Authors & Referees and the Editorial Policy Checklist.

Statistical parameters

When statistical analyses are reported, confirm that the following items are present in the relevant location (e.g. figure legend, table legend, main text, or Methods section).

n/a	Confirmed	
☐	☒	The exact sample size (*n*) for each experimental group/condition, given as a discrete number and unit of measurement
☐	☒	An indication of whether measurements were taken from distinct samples or whether the same sample was measured repeatedly
☐	☒	The statistical test(s) used AND whether they are one- or two-sided<br>*Only common tests should be described solely by name; describe more complex techniques in the Methods section.*
☒	☐	A description of all covariates tested
☒	☐	A description of any assumptions or corrections, such as tests of normality and adjustment for multiple comparisons
☐	☒	A full description of the statistics including central tendency (e.g. means) or other basic estimates (e.g. regression coefficient) AND variation (e.g. standard deviation) or associated estimates of uncertainty (e.g. confidence intervals)
☒	☐	For null hypothesis testing, the test statistic (e.g. *F*, *t*, *r*) with confidence intervals, effect sizes, degrees of freedom and *P* value noted<br>*Give P values as exact values whenever suitable.*
☒	☐	For Bayesian analysis, information on the choice of priors and Markov chain Monte Carlo settings
☒	☐	For hierarchical and complex designs, identification of the appropriate level for tests and full reporting of outcomes
☒	☐	Estimates of effect sizes (e.g. Cohen's *d*, Pearson's *r*), indicating how they were calculated
☐	☒	Clearly defined error bars<br>*State explicitly what error bars represent (e.g. SD, SE, CI)*

Our web collection on statistics for biologists may be useful.

Software and code

Policy information about availability of computer code

Data collection	EPU (Fisher), Tecan-iControl, Peak Chart (Brandel)
Data analysis	MotionCor2, CTFFIND4, ReLion, Pymol, UCSF Chimera, Resmap, Coot, Molprobity, CNS, Excel, EMAN2, Swiss Modeler, IUPred, Clustal Omega, JalView

For manuscripts utilizing custom algorithms or software that are central to the research but not yet described in published literature, software must be made available to editors/reviewers upon request. We strongly encourage code deposition in a community repository (e.g. GitHub). See the Nature Research guidelines for submitting code & software for further information.

Data

Policy information about availability of data

All manuscripts must include a data availability statement. This statement should provide the following information, where applicable:

- Accession codes, unique identifiers, or web links for publicly available datasets
- A list of figures that have associated raw data
- A description of any restrictions on data availability

All data and constructs used in this study will be made available upon request. EM maps and atomic coordinates for the cryo-EM model have been deposited in the EMDB and PDB with accession codes EMDB-9063 and PDB 6MAT. The source data underlying Fig. 1b, Fig. 4e, Fig. 6C, and Fig 6f are provided as a Source Data File.

Field-specific reporting

Please select the best fit for your research. If you are not sure, read the appropriate sections before making your selection.

☒ Life sciences ☐ Behavioural & social sciences ☐ Ecological, evolutionary & environmental sciences

For a reference copy of the document with all sections, see nature.com/authors/policies/ReportingSummary-flat.pdf

Life sciences study design

All studies must disclose on these points even when the disclosure is negative.

Sample size	The 3D reconstruction was calculated from thousands of images.
Data exclusions	During cryo-EM data processing poor particle images were discarded following 2D and 3D classification.
Replication	All experiments (growth curves and sucrose gradients) were performed in triplicate.
Randomization	N/A
Blinding	N/A

Reporting for specific materials, systems and methods

Materials & experimental systems

n/a	Involved in the study	
☐	☒	Unique biological materials
☐	☒	Antibodies
☐	☒	Eukaryotic cell lines
☒	☐	Palaeontology
☒	☐	Animals and other organisms
☒	☐	Human research participants

Methods

n/a	Involved in the study	
☒	☐	ChIP-seq
☒	☐	Flow cytometry
☒	☐	MRI-based neuroimaging

Unique biological materials

Policy information about availability of materials

Obtaining unique materials	All data and constructs used in this study will be made available upon request.

Antibodies

Antibodies used	Anti-Flag (Sigma-Aldrich, #f7425), Anti-alpha Tubulin Yol1/34(Abcam, #ab6161), Goat anti-rat IgG (R&D Systems, #HAF005), Goat anti-rabbit IgG (Sigma-Aldrich, #A0545)
Validation	The anti-Flag antibody was verified with an untagged yeast strain.

Eukaryotic cell lines

Policy information about cell lines

Cell line source(s)	The Rix7 tetO7 strain was obtained from GE Dharmacon. All other strains will be made available upon request.
Authentication	We verified that the original Rix7 tetO7 strain was correct by carrying out growth curves in the presence of DOX.
Mycoplasma contamination	Cell lines were not tested for mycoplasma contamination.
Commonly misidentified lines (See ICLAC register)	No commonly misidentified lines were used in this study.
